# The Effect of Prolyl Isomerase Pin1 on the Development of Metabolic Dysfunction-Associated Steatohepatitis and a New Treatment Strategy

**DOI:** 10.3390/ijms27177788

**Published:** 2026-08-31

**Authors:** Yasuka Matsunaga, Masa-Ki Inoue, Machi Kanna, Tomoichiro Asano, Yusuke Nakatsu

**Affiliations:** 1 John W. Deming Department of Medicine, Tulane University School of Medicine, New Orleans, LA 70112, USA; ymatsunaga@tulane.edu; 2 Division of Nephrology, Department of Medicine, Vanderbilt University Medical Center, Nashville, TN 37232, USA; masaki.inoue@vumc.org; 3 Department of Mechanical Engineering, Faculty of Engineering, Sanyo-Onoda City University, Daigakudori 1-1-1, Sanyo-Onoda 756-0884, Japan; kanna@rs.socu.ac.jp; 4 Department of Biomedical Chemistry, Graduate School of Biomedical and Health Sciences, Hiroshima University, Hiroshima 734-8553, Japan; tasano@hiroshima-u.ac.jp

**Keywords:** Pin1, MASLD, MASH, inhibitors, PPARα, ATGL, PRDM16

## Abstract

Metabolic dysfunction-associated steatohepatitis (MASH) is characterized by fatty liver, inflammation, and fibrosis, which eventually results in hepatocarcinoma. Recently, MASH therapeutics have been approved by the FDA; however, options remain limited, and the discovery of additional molecular targets is ongoing. The prolyl isomerase Pin1 regulates the function of phosphorylated substrates by converting the *cis*-*trans* form of proline. Pin1 interacts with specific substrates in each organ and controls lipid and energy metabolism through modulating the localization and stabilization of target proteins. Interestingly, a high-calorie diet upregulates Pin1 expression in the liver and adipocytes. It suppresses fatty acid oxidation, thermogenesis, and lipolysis by regulating PPARα, PRDM16, and adipose triglyceride lipase, which ultimately leads to steatosis. Beyond metabolic dysregulation, Pin1 is essential for activating hepatic stellate cells, which cause liver fibrosis. Pin1 conditional knockout mice exhibit resistance to high-calorie diet-induced MASH, which indicates a role for Pin1 in multiple organs as a central regulator linking metabolic dysfunction to liver inflammation and fibrosis. These results suggest that Pin1 inhibitors may be effective for MASH treatment. In this review, we discuss the roles of Pin1 in MASH development and as a potential therapy target.

## 1. Metabolic Dysfunction-Associated Steatotic Liver Disease/Metabolic Dysfunction-Associated Steatohepatitis

Metabolic dysfunction-associated steatotic liver disease (MASLD) has emerged as an overarching clinical entity that encompasses the full spectrum of hepatic steatosis. MASLD is formally defined by the presence of macrovesicular steatosis in >5% of hepatocytes in individuals with at least one metabolic risk factor, in the absence of excessive alcohol consumption or other secondary etiologies [1]. Epidemiological data indicate that MASLD affects 25–40% of the adult population worldwide [2,3], with roughly 16% of these cases transitioning to metabolic dysfunction-associated steatohepatitis (MASH) [3]. MASH represents a more aggressive histological phenotype. It is distinguished by lobular inflammation and hepatocellular ballooning, which significantly increases the risk of progressive liver damage, cirrhosis, and hepatocellular carcinoma [1,4]. Recent longitudinal studies highlight a concerning rise in the prevalence of clinically significant fibrosis (stage F2 or higher), which solidifies MASH as a global public health priority [5]. Moreover, MASLD is intrinsically linked with systemic metabolic dysregulation, including insulin resistance, dyslipidemia, central obesity, and hypertension, and often serves as a pivotal hepatic manifestation of these multisystemic disorders [6,7].

The fundamental drivers of MASLD and MASH pathogenesis are localized and systemic insulin resistance, which primarily arise from chronic excess caloric intake and the subsequent failure of adipose tissue to expand sufficiently to store surplus lipids [8,9]. This metabolic imbalance results in the accumulation of triglycerides in the liver and accelerates de novo lipogenesis (DNL) [9]. Historically, a “two-hit hypothesis” was the predominant framework, which suggests that initial steatosis sensitizes the liver to subsequent inflammatory insults [10]; however, this model has been superseded by the “multiple-parallel hits” hypothesis. This contemporary paradigm proposes that a complex interplay of nutritional factors, gut microbiota dysbiosis, genetic predisposition, and pro-inflammatory cytokine signaling occurs simultaneously to drive the progression from simple steatosis to necroinflammation and the fibrogenesis characteristics of MASH [11].

Despite our increased understanding of its molecular basis, MASH treatment remains lifestyle modification through dietary intervention and physical activity [1]. Nevertheless, achieving a clinically validated threshold of >10% weight loss within a 1- to 2-year period remains a formidable challenge for patients, with only 10–20% successfully achieving and maintaining this goal [12,13]. This has fueled the development of pharmacological agents. Resmetirom, a thyroid hormone receptor-β (THR-β) selective agonist, recently became the first specific therapy approved by the FDA for treating non-cirrhotic MASH with significant fibrosis (F2-F3) [14]. In addition, the GLP-1 receptor agonist semaglutide has received FDA approval for ameliorating liver fibrosis [15]. However, it is reported that Resmetirom improves fibrosis inonly 20~30% patients [14]. In addition, the effects of semaglutide on fibrosis could be limited, and gastrointestinal adverse effects are frequently observed [16,17].

Accordingly, definitive and widely accessible treatment options for MASH are still limited, which highlights the necessity for continued clinical studies.

## 2. Prolyl Isomerase Pin1

**Figure 1:**
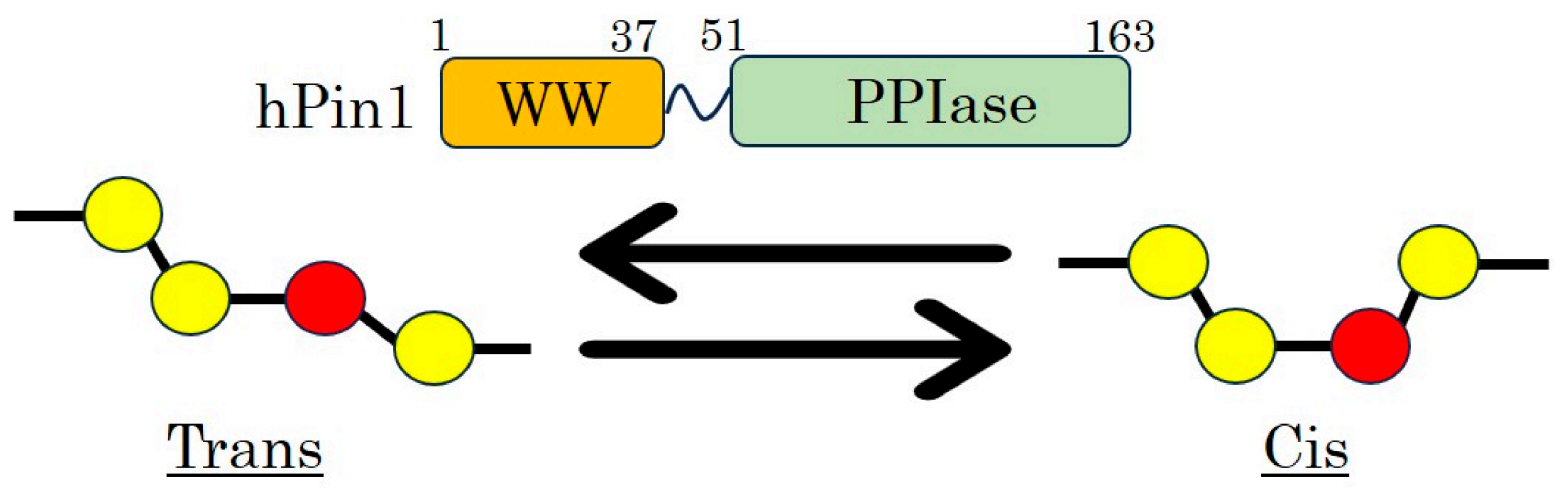
Pin1 catalyzes the isomerization of cis-trans. Yellow circle represents amino acids, and red circle indicates proline. The arrows show Pin1-catalyzed conversion of proline.

Prolyl isomerase Pin1 was initially isolated as a factor that interacts with NIMA during cell division [18]. Subsequent studies revealed that Pin1 typically binds to serine/threonine phosphorylation sites (pSer/pThr-Pro-motifs) (Figure 1) [19]. By catalyzing the cis-trans isomerization of proline residues, Pin1 regulates various aspects of target protein function, including protein stability, subcellular localization, and post-translational modifications [20]. Importantly, the activity and function of Pin1 itself are regulated through post-translational modification [21,22,23,24,25,26]. For example, Pin1 phosphorylation by polo-like kinase 1 (PLK1) enhances its protein stability, whereas modification by death-associated protein kinase (DAPK) suppresses its enzymatic activity [22,23]. These regulatory modifications suggest a mechanistic link between Pin1 dysregulation and disease pathogenesis.

Pin1 also plays a role in cancer etiology and progression [27]. Its expression is upregulated in a wide range of human malignancies, and genetic or pharmacological inhibition of Pin1 markedly suppresses tumor growth [28,29]. Besides its oncogenic functions, recent studies have implicated Pin1 in the pathogenesis of metabolic disorders, including obesity, MASLD, and metabolic dysfunction-associated steatohepatitis (MASH) [30,31,32].

Because Pin1 interacts with a variety of targets, its inhibition can simultaneously modulate multiple pathways involved in the pathogenesis of MASLD/MASH, including lipogenesis, fatty acid oxidation and inflammation. Indeed, Pin1-deficient mice are resistant to the development of MASLD/MASH, and Pin1 deletion ameliorates steatosis, inflammation, and fibrosis. These results indicate that Pin1 is a key pathogenic driver in the progression of metabolic liver diseases, including MASLD/MASH [33,34].

In rodent models, a high-calorie diet increases Pin1 protein expression in multiple metabolic tissues, including the liver, adipose tissue, and pancreatic islets [30,32,35]. Consistent with these findings, Pin1 staining of liver biopsy specimens from MASH patients revealed strong nuclear Pin1 expression [36]. Although the mechanisms underlying the increase in Pin1 expressionare not completely understood, the upregulation of protein stability by post-translational modification likely contributes. MASLD/MASH livers are characterized by chronic inflammation and sustained activation of c-Jun N-terminal kinase (JNK), which phosphorylates Pin1 at Ser115 to enhance its protein stability [24]. In addition, PLK1 and the deubiquitinating enzyme USP34 may also contribute to increased hepatic Pin1 protein stability [22,25].

Serum/Plasma Pin1 concentrations are significantly increased in patients with MASH compared with healthy controls, and these levels are positively correlated with fibrosis stage [37]. Another report also showed that plasma concentrations in patients with MASH are higher than in healthy volunteers [38]. In addition, peripheral blood monocytes prepared from type 2 diabetes patients reveal higher Pin1 expressions and activity, compared to healthy controls [39]. While, Pin1 concentrations in the serum of young people with obesity were comparable with lean controls [40]. Thus, although alterations of Pin1 in young people with MASH are poor, the detection of Pin1 in adults with MASH could be beneficial as a diagnostic biomarker (Table 1). Overall, increased Pin1expression, including that observed in human livers, could contribute to the development and progression of MASLD/MASH by regulating multiple target proteins. Therefore, Pin1 inhibitors could represent a promising therapeutic strategy for MASLD/MASH. 

## 3. The Roles of Pin1 in Steatosis

### 3.1. Lipogenesis and Fatty Acid Oxidation

The accumulation of hepatic lipids is considered an early and fundamental event in the development of MASLD/MASH [7]. Under normal physiological conditions, lipid homeostasis is tightly regulated in hepatocytes through a balance between lipogenesis and fatty acid oxidation (FAO) [41]. In MASH, this regulatory balance is frequently disrupted, which is characterized by enhanced lipogenesis and suppressed FAO. This metabolic dysregulation results in excessive lipid deposition in the liver, thereby promoting hepatic steatosis and contributing to MASH progression.

Pin1 in hepatocytes promotes fatty acid accumulation. Hepatocyte-specific Pin1 knockout (H-Pin1 KO) mice are resistant to hepatic steatosis when fed a high-fat, high-cholesterol (HFHC) diet [30]. Consistently, global Pin1 deficiency ameliorates methionine–choline-deficient (MCD) diet-induced MASH [33,34].

Mechanistically, Pin1 interacts with acetyl-CoA carboxylase 1 (ACC1), a rate-limiting enzyme in the lipogenesis pathway, and prevents ACC1 degradation by autophagy, although Pin1 does not affect ACC mRNA expression [42]. This stabilizes its protein levels, thereby promoting lipogenesis [34,42]. In addition, during obesity, autophagy in the liver is impaired [43], which increases ACC protein levels.

Sterol regulatory element–binding protein (SREBP1) is a master regulator that induces lipogenic gene expression. Although the interaction between Pin1 and SREBP1 is unclear, Pin1 deletion in hepatocytes downregulates the expression of lipogenic genes, which suggests that Pin1 enhances SREBP1 transcriptional activity [30]. Interestingly, Pin1 is revealed to interact with SREBP2 which is a master regulator of cholesterol metabolism. Pin1 upregulates the protein levels of pre- and mature-SREBP2 [29]. Based on these facts, Pin1 may stabilize SREBP1 protein levels through interactions similar to those of SREBP2. Alternatively, Pin1 may interact with the transcriptional factor ChREBP, which induces lipogenesis-related genes and upregulates its functions. 

Pin1 also associates with peroxisome proliferator-activated receptor α (PPARα) and suppresses its transcriptional activity, which results in reduced FAO [30]. Moreover, H-Pin1 KO mice exhibit higher concentrations of beta-hydroxybutyrate (BHB), which is produced by FAO [30]. Although the mechanism by which Pin1 decreases PPARα activity remains unclear, Pin1 may disrupt the association between PPARα and co-transcriptional factors, such as PGC-1α. Alternatively, Pin1 may disrupt the binding of PPARα to DNA. 

In addition to PPARα signaling, AMP-activated protein kinase indirectly promotes FAO and limits lipid accumulation [44]. It phosphorylates ACC and inactivates its function, resulting in a decrease in malonyl CoA which inhibits CPT-1. Pin1 interacts with the γ subunit of AMPK and was shown to decrease AMPKα phosphorylation in 293T and HepG2 cells [45]. In vivo, however, the role of AMPK in Pin1-mediated metabolic regulation appears to be model-dependent. In the HFHC diet model, Pin1 deficiency does not affect hepatic AMPK phosphorylation; however, in the MCD diet model, AMPK and ACC phosphorylation are significantly increased in the livers of Pin1 knockout mice [30,40].

In addition to its role in lipid metabolism, Pin1 may contribute to MASH development through the regulation of methionine adenosyltransferase α1 (MATα1). Pin1 prevents the translocation of MATα1 to the mitochondria, resulting in disrupted mitochondrial function, such as β-oxidation and oxidative phosphorylation [46].

**Figure 2:**
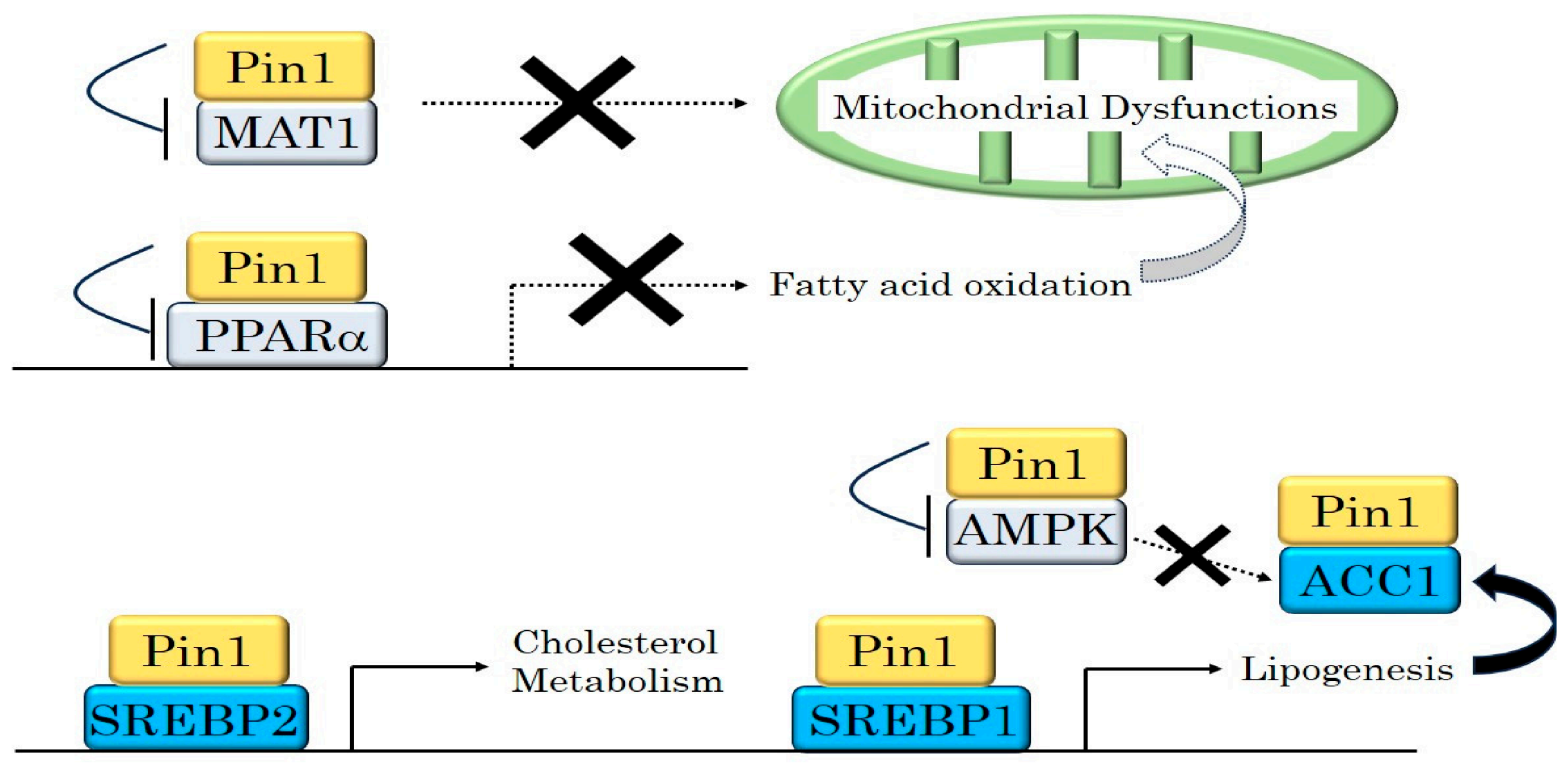
Pin1 in hepatocytes causes MASLD/MASH. Pin1 enhances lipogenesis by stabilizing ACC1. This leads to mitochondrial dysfunction, including fatty acid oxidation, through its interaction with MAT1α and PPARα.

Taken together, these Pin1-mediated modifications disrupt the balance of hepatic lipid metabolism, thereby promoting lipid accumulation and steatosis (Figure 2).

### 3.2. Hyperinsulinemia

Hyperinsulinemia and insulin resistance are frequently observed in MASLD [47]. In the liver, insulin normally suppresses gluconeogenesis, while promoting lipogenesis; however, hepatic insulin resistance selectively impairs the inhibition of gluconeogenesis, whereas insulin-driven lipogenesis remains largely preserved [48]. As a result, chronic hyperinsulinemia may play an important role in the development of hepatic steatosis.

Pin1 is a positive regulator of pancreatic β-cell function, and a high-fat diet increases Pin1 expression in pancreatic islets. Notably, Pin1 enhances glucose-stimulated insulin secretion by modulating the SIK2–CDK5–p35 signaling pathway. Pin1 directly binds to SIK2 and enhances its activity. Subsequently, SIK2 phosphorylates p35 and induces its degradation, canceling the repression of CDK5 for the voltage-dependent calcium channel [35]. In addition, it contributes to compensatory β-cell proliferation through the stabilization of cyclin D1 [35,49].

Pin1 promotes insulin signaling by upregulating the tyrosine phosphorylation of insulin receptor substrate 1 (IRS-1) [50]. Thus, Pin1 deficiency exacerbates insulin resistance, although H-Pin1 KO mice exhibit insulin sensitivity [30]. The reduction in lipid accumulation in the liver via Pin1 deficiency may offset impaired insulin signaling. 

Taken together, Pin1 activity in pancreatic β cells may promote hyperinsulinemia and contribute to the development of fatty liver during obesity.

## 4. Inflammation

Inflammation triggers the transition from MASLD to MASH. Inflammatory cytokines damage the liver, and these injuries lead to liver fibrosis [51]. NF-κB is a key transcription factor involved in the induction of inflammatory cytokines [52]. NF-κB consists of p65 and p50 subunits, and is sequestered in the cytosol through interaction with IκB. Upon stimulation, such as by lipopolysaccharide (LPS), IκB is phosphorylated and subsequently degraded. As a result, the p65–p50 complex translocates from the cytosol to the nucleus, where it functions as a transcription factor [53].

Pin1 associates with p65 through a motif of pThr254-Pro, and promotes both its protein stability and nuclear translocation. In addition, this interaction enhances p65 phosphorylation at Thr254 and Ser276 sites [54,55]. 

AP-1 is another key factor in inflammatory responses. Pin1 indirectly upregulates AP-1 activity through its interaction with p70 S6K. Pin1 upregulates the phosphorylation of p70S6K and subsequently ERK activation. These results eventually lead to the potentiation of AP-1 transcriptional activity [56]. 

Moreover, Pin1 is involved in promoting activation of the NLRP inflammasome. Indeed, Pin1-deficient bone marrow-derived macrophages show reduced secretion of IL-1β and IL-18 compared to wild-type cells. Mechanistically, Pin1 binds to p38 MAPK and enhances its phosphorylation. This phosphorylation leads to increased expression of ASC and NLRP3, which are components of the inflammasome [57]. 

Taken together, Pin1 potently promotes inflammation by regulating multiple substrates.

## 5. The Impact of Adipose Pin1 on Obesity and MASH Development

Excess lipid accumulation in the liver is strongly linked to adipose tissue dysfunction. Hepatic fatty acids are primarily derived from adipose tissue. Insulin is the principal hormonal regulator responsible for suppressing lipolysis in adipocytes, whereas insulin resistance in adipose tissue results in dysregulated lipolysis. [58]. Adipose tissue dysfunction may represent the predominant pathogenic mechanism in a subset of individuals presenting with lean MASH [8].

Adipocyte-specific Pin1-deficient mice have lower body weights with liver triglyceride and macrophage infiltration of epididymal white adipose tissue during diet-induced obesity [31]. Pin1 directly interacts with adipose triglyceride lipase (ATGL), the rate-limiting enzyme of triglyceride lipolysis. It reduces lipolysis activity through ATGL degradation and promotes glucose tolerance and inflammation in adipose tissue [31]. Mechanistically, Pin1 may enhance the recruitment of the E3 ligase COP1 to ATGL and promote ATGL degradation. Interestingly, adipocyte-specific Pin1 deficiency ameliorates glucose and insulin tolerance and fatty liver development, indicating that improved adipose function is closely associated with protection against MASH [31].

Thermogenesis in adipocytes consumes excessive nutrients as heat. Accordingly, the impairment of thermogenesis decreases energy expenditure, and eventually, leads to the accumulation of lipids in the liver or adipocytes. Pin1 plays an important role in the regulation of non-shivering thermogenesis in brown and beige adipocytes. It directly interacts with PR domain-containing 16 (PRDM16), a master regulator of uncoupling protein (UCP-1) that contributes to thermogenic programs. Pin1 facilitates PRDM16 degradation through the ubiquitin–proteasome system in an isomerase-dependent manner. As CUL2 is an E3 ligase for PRDM16, Pin1 may promote their association, thereby decreasing its protein levels. Consistent with these findings, Pin1 in adipocytes upregulates thermogenic genes and increases oxygen consumption [32]. Generally, the increase in free fatty acids by lipolysis causes fatty liver; however, adipocyte-specific Pin1 KO mice reveal a reduction in lipid accumulation in the liver under high-fat diet conditions. This occurs because free fatty acids generated in white adipose tissue are consumed for heat generation in beige and brown adipocytes.

In addition to regulating lipid metabolism, Pin1 enhances adipocyte differentiation by peroxisome proliferator-activated receptor γ (PPARγ) protein in adipocytes by upregulating its transcriptional activity [59]. PPARγ expression increases during adipocyte differentiation, whereas Pin1 knockdown impairs differentiation in 3T3-L1 cells [59]. Moreover, Pin1 may affect mitotic clonal expansion, which is an important early step in adipogenesis. Regulation of PKM2 by Pin1 may be responsible. Pin1 recognizes the S37 site in PKM2 and enhances the translocation of PKM2 to the nucleus [60]. Moreover, PKM2 knockdown in preadipocyte 3T3-L1 cells impairs adipogenesis [61]. Taken together, these results indicate that PKM2 regulation by Pin1 is essential for adipogenesis. In sup-port of this, Pin1 has been shown to promote cell cycle progression in various cell types [18].

**Figure 3:**
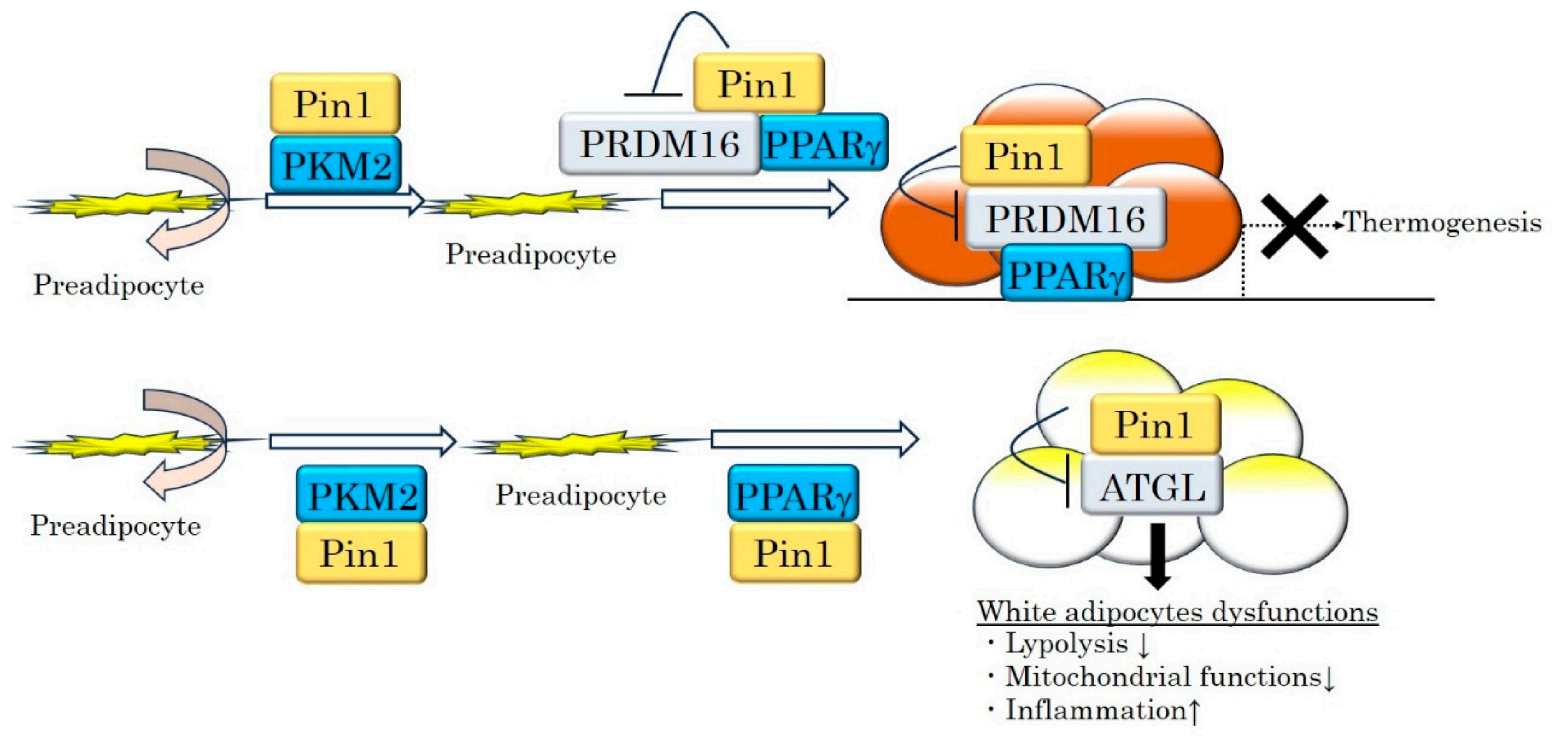
Pin1 in adipocytes affects the onset of MASLD/MASH. Pin1 impairs lipolysis and mito-chondrial function through ATGL degradation. In brown adipocytes, Pin1 suppresses thermogene-sis by downregulating PRDM16.

Collectively, Pin1 is a key regulator of adipose tissue dysfunction and contributes significantly to the development of MASH (Figure 3).

## 6. The Roles of Pin1 in Fibrosis

Hepatic stellate cells (HSCs) play an important role in liver fibrogenesis, which is tightly coupled to profound metabolic reprogramming [62]. Upon activation, HSCs undergo a metabolic shift that includes excessive extracellular matrix (ECM) production, largely driven by transforming growth factor-β (TGF-β). The Smad pathway primarily mediates TGF-β signaling. Smad2/3 is phosphorylated by TGF-β receptor I, which translocates to the nucleus to regulate fibrogenic gene expression in cooperation with multiple transcriptional partners [63].

Pin1 is a key regulator linking TGF-β/Smad signaling to metabolic control in HSCs. Pin1 knockdown markedly suppresses TGF-β–induced ECM production, including collagen expression, in LX-2 cells [64,65]. Pin1 interacts with Smad3 and enhances its transcriptional activity [60]. It further promotes an association between Smad3 and the transcriptional co-factor TAZ, thereby amplifying the Smad-dependent transcription of profibrotic genes [65]. As Smad3 has a variety of binding partners, Pin1 may change the components of Smad complexes, in addition to TAZ. Moreover, Pin1 binds to Smad6, an inhibitory Smad, and promotes its nuclear retention, thereby attenuating its suppressive effect on TGF-β/Smad signaling [66]. This may result from Pin1 interrupting the association between Smad6 and exportin. Indeed, Pin1 was reported to disrupt delivery through xportin-5 [67]. Furthermore, Pin1 stabilizes TGF-β mRNA, reinforcing its sustained profibrotic signaling [68].

Autophagy-dependent lipid utilization is another metabolic pathway essential for HSC activation. Autophagy contributes to the degradation of lipid droplets, which provides energy substrates required for the activated phenotype of HSCs [69]. Pin1 regulates autophagy by interacting with Foxo3 and inhibiting its phosphorylation. This leads to the nuclear accumulation of Foxo3 and transcriptional induction of autophagy-related genes [70]. This suggests that Pin1-driven autophagy may support the metabolic demands of activated HSCs.

**Figure 4:**
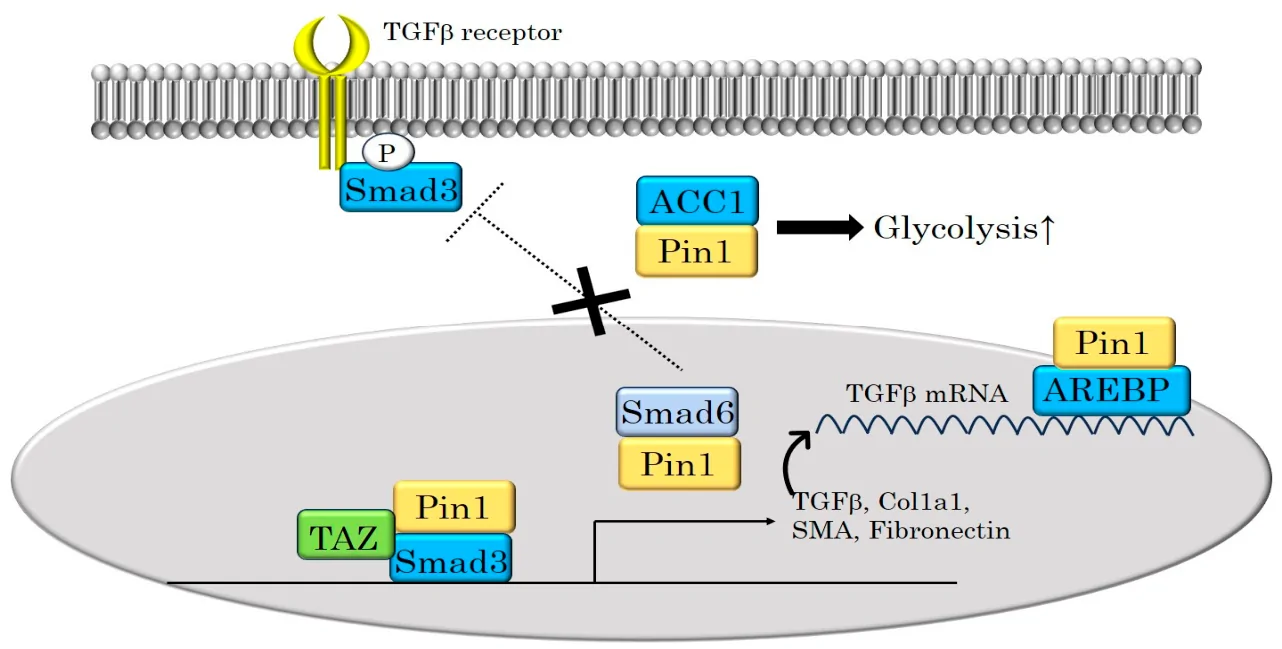
Pin1 is essential for activating HSCs. Pin1 stabilizes TGFβ mRNA and enhances Smad signaling. Pin1 enhances the interaction between Smad3 and TAZ, which upregulates their transcriptional activity. Moreover, Pin1 binds to Smad6 and suppresses its inhibitory effects. AREBP: AU-rich elements binding protein.

Lipid metabolism is also involved in HSC activation. Enhanced lipogenesis mediated by acetyl-CoA carboxylase 1 (ACC1) promotes HSC activation, whereas pharmacological inhibition of ACC1 suppresses this process [71]. Because Pin1 stabilizes ACC1 protein expression [30], Pin1-mediated upregulation of ACC1 may facilitate lipogenic reprogramming and contribute to HSC activation in LX-2 cells. Taken together, Pin1 is a central metabolic regulator in HSCs by coordinating TGF-β/Smad signaling, autophagy-mediated energy supply, and lipid metabolic remodeling, thus promoting HSC activation and liver fibrogenesis (Figure 4).

## 7. Pin1 May Contribute to the Transition from MASH to Hepatocarcinoma

MASH is known to progress to hepatocellular carcinoma (HCC) in some cases. Notably, Pin1 expression is upregulated in HCC, and Pin1 plays a significant role in hepatocarcinogenesis [72].

Although the mechanisms underlying the transition from MASH to HCC remain unclear, alterations in Pin1 expression may be involved. A high-calorie diet has been shown to increase Pin1 protein levels in the liver, leading to lipid accumulation, inflammation, and liver injury. These pathological changes stimulate compensatory cell proliferation for tissue repair. In addition, danger-associated molecular patterns (DAMPs) released from dead or damaged cells may further enhance Pin1 expression.

This sustained elevation of Pin1 may promote excessive cell proliferation while suppressing apoptosis. Together, these effects could contribute to the initiation and progression of HCC.

Collectively, these findings suggest that Pin1 is a key factor in the progression of MASH to HCC.

## 8. Development of Pin1 Inhibitors to Treat Metabolic Syndromes

Because the deletion of Pin1 ameliorates the symptoms of metabolic syndromes, including MASLD/MASH, Pin1 inhibitors may represent effective therapeutic compounds.

Indeed, the inhibition of Pin1 ameliorates metabolic dysfunction in various rodent models [73,74]. In this section, we introduce and discuss the potential of representative Pin1 inhibitors (Table 2).

### 8.1. Juglone

Juglone is one of the most extensively studied Pin1 inhibitors, and suppresses Pin1 isomerase activity. Although its efficacy in directly reversing metabolic syndromes is unclear, juglone attenuates dimethylnitrosamine-induced liver fibrosis [73]. Furthermore, it has protective effects against both cardiac and hepatic fibrosis, indicating a potential role for Pin1 inhibition in fibrotic diseases, including MASH [73].

Because juglone inhibits Pin1 activity by interacting with the thiol group of cysteine residues, its selectivity is doubtful, and it may attack unpredictable targets [75]. Moreover, juglone generates reactive oxygen species [76]. Thus, it remains unclear whether all of its observed effects result entirely from the selective inhibition of Pin1.

### 8.2. Epigallocatechin Gallate

Epigallocatechin gallate (EGCG) is another well-known Pin1 inhibitor that improves key pathological features of metabolic dysfunction-associated steatohepatitis (MASH), including hepatic steatosis and activation of HSCs [74,77]. Interestingly, EGCG associates with the WW and PPIase domains in Pin1 [78]. Based on this feature, EGCG can prevent the interaction between Pin1 and its substrates, in addition to inhibiting isomerase activity. EGCG acts on the 67k Da laminin receptor (67LR) and promotes subsequent signaling [79], indicating that EGCG exerts its protective effects on MASH through Pin1-dependent and -independent pathways. Because EGCG is a component of green tea and is considered relatively safe, it represents a promising therapeutic agent for treating MASH.

### 8.3. All Trans-Retinoic Acid and Arsenic Trioxide

All trans-retinoic acid (ATRA) which is a drug for acute promyelocytic leukemia (APL) was found to be a Pin1 inhibitor by screening [80]. In contrast to other Pin1 inhibitors, ATRA facilitates the degradation of Pin1 through the ubiquitin–proteasome pathway. [80]. ATRA is also a non-selective Pin1 inhibitor that affects c-mer tyrosine kinase (MerTK) activity, which promotes liver fibrosis [81]. ATRA causes MerTK cleavage through p38-ADAM17, which ameliorates liver fibrosis in MASH model mice. Thus, the administration of ATRA to mice improves MASH symptoms by targeting Pin1 and MerTK.

Arsenic trioxide (ATO) is also used in combination with ATRA for APL treatment [82]. Interestingly, ATO causes Pin1 degradation, similar to ATRA. Although it is unclear whether ATO relieves MASH symptoms, ATO is similar to ATRA, which is also expected to improve MASH.

Although ATRA and ATO are anticancer drugs, their side effects cannot be ignored. Accordingly, long-term administration for MASH treatment may be inadequate, and a system that selectively delivers drugs to HSC or Kupffer cells may be essential.

### 8.4. API-1

API-1, which targets the isomerase domain of Pin1, inhibits the proliferation and migration of hepatocellular carcinoma cells [83]. Although the metabolic effects of API-1 have not yet been fully elucidated, it was reported to alleviate the activation of HSCs [65].

As a limitation, API-1 has low water solubility, resulting in low bioavailability in vivo [84]. To resolve this issue, API-1 wrapped with nanoscale liposomes (API-LP) has been developed. As expected, API-LP showed higher antitumor effects compared with API-1. Importantly, the administration of API-LP to mice caused no apparent toxicity in some tissues, including the liver [84].

Considering these facts, API-LP may exert antifibrotic effects in MASH model mice without toxicity.

### 8.5. KPT-6566

KPT-6566 is a Pin1 inhibitor with unique activity [85]. It covalently binds to Pin1 and induces its degradation. After binding, part of the KPT-6566 structure is cleaved, and a quinone-mimicking byproduct is produced. This byproduct generates reactive oxygen species and induces cell death [85].

When considered as a drug for MASH, its toxicity could be a major issue. KPT-6566 covalently binds to the PPIase domain and degrades Pin1. Its derivatives, which produce no byproducts, are considered attractive compounds for MASH treatment.

### 8.6. PROTAC

A proteolysis-targeting chimera (PROTAC) is a novel compound possessing two active domains. By binding simultaneously to a target protein and an E3 ubiquitin ligase to form a ternary complex, it promotes ubiquitination and thereby induces degradation of the target protein. Due to these characteristics, PROTACs are known for their high target specificity and low cytotoxicity [86,87].

In addition to small-molecule inhibitors, a Pin1-targeting PROTAC degrader, P1D-34, was recently developed [88]. Unlike API-1 or EGCG, which do not alter Pin1 protein abundance, P1D-34 induces Pin1 degradation in a concentration-dependent manner [88]. 

As Pin1 regulates some substrates through a PPIase-independent mechanism, the degradation of Pin1 may diminish its role as a scaffold protein and lead to more effective therapy.

Although the safety and bioavailability of PROTACs need to be investigated in the future, this provides an alternative and potentially more effective approach to modulating Pin1 function in metabolic and fibrotic diseases.

## 9. Summary of Pin1 as an Attractive Target for MASLD/MASH

Pin1 has important roles in lipid and energy metabolism in a variety of organs. Overall, it promotes energy accumulation, including lipid storage, by regulating multiple proteins. Consequently, under conditions of sustained overnutrition, Pin1 contributes to the development of MASLD/MASH that accompanies obesity.

Pin1 inhibitors may serve as therapeutic agents for MASLD/MASH. Inhibition of Pin1 is expected to have several beneficial effects as follows: (1) downregulation of lipogenesis through a reduction in ACC protein levels; (2) upregulation of FAO via activation of PPARα; (3) suppression of HSC activation through impaired Smad signaling and cellular energy metabolism; and (4) improvement of adipocyte function.

Evidence of alterations in Pin1 expression in human tissues remains limited. Furthermore, studies using tissue-specific Pin1 knockout (KO) mice are lacking. For example, the phenotypes of HSC- or macrophage-specific Pin1 KO mice have yet to be examined, and the physiological role of Pin1 in these cell types in vivo remains largely unknown. Addressing these questions will be essential for clarifying the tissue-specific functions of Pin1 and evaluating the therapeutic potential of Pin1-targeted interventions in MASH.

Several Pin1 inhibitors have shown therapeutic efficacy in rodent models of metabolic syndrome; however, there are concerns regarding the clinical use of Pin1 inhibitors.

As most of the current Pin1 inhibitors have been developed as anticancer drugs, their toxicity is a major issue. Although juglone and KPT-6566 exert strong anticancer effects, they generate ROS and may harm normal cells. Some compounds, such as EGCG or API-1, have issues with bioavailability.

In addition, because Pin1 is ubiquitously expressed, systemic inhibition may result in adverse side effects. Some studies suggest that Pin1 exerts multifaceted effects on tau pathology, a key feature of Alzheimer’s disease. Specifically, Pin1 suppresses tau accumulation in wild-type tau models, and Pin1 KO mice exhibit abnormal behavior in old age [89]. Moreover, Pin1 deficiency affects germ cell function. Pin1 is essential for the progression of oocyte and sperm production [90,91]. Another concern is that Pin1 inhibition may cause senescence, as it promotes the cell cycle. These findings may represent a limitation to the long-term use of Pin1 inhibitors.

These issues may be addressed through the development of targeted drug delivery systems. For example, a vitamin A-modified liposome could be beneficial for targeting HSCs, as HSCs have the characteristicof vitamin A uptake. Delivery tools to the hepatocyte, such as N-acetylgalactosamine, may also be beneficial. Targeted delivery of relatively selective compounds, such as API-1, to hepatocytes or hepatic stellate cells via a drug delivery system (DDS) represents an effective therapeutic strategy.

Recent clinical success of PROTAC technology has demonstrated the feasibility of targeted protein degradation in humans. Although Pin1-targeting PROTACs remain in a preclinical stage, this therapeutic modality may overcome some limitations of conventional Pin1 inhibitors by eliminating the enzymatic and scaffold functions of Pin1; however, further optimization of tissue specificity, bioavailability, and long-term safety will be required before clinical application in chronic diseases, such as MASH.

In conclusion, Pin1 plays an important role in the development of MASLD/MASH. The development of selective Pin1 inhibitors represents a promising therapeutic strategy for this disease.

## Figures and Tables

**Table 1:** The alterations of Pin1 in patients with metabolic syndromes.

Disease	Samples	Changes in Pin1, Compared to Healthy Control	References
MASH	Liver biopsy	Strong staining	[36]
MASH	Blood serum	High concentrations	[37]
MAFLD	Blood plasma	High concentrations	[38]
Type2 Diabetes	Peripheral blood monocytes	High mRNA levels and activity	[39]
Obese	Blood serum	No differences	[40]

**Table 2:** Summary of Pin1 inhibitors.

Inhibitors	Effects on Pin1	Pin1-Independent Effects	Other Features
Juglone	Inhibition of activity	ROS generation	
EGCG	Inhibition of activity Prevention of interaction with substrates	Promotion of 67LR signaling	
ATRA	Decrease of Pin1 protein	Effects on MerTK activity Degradation of PML-RARa	Approved as a treatment for APL
ASO	Decrease of Pin1 protein	Degradation of PML-RARa	Approved as a treatment for APL
API-1	Inhibition of activity		Low bioavailability
KPT-6566	Decrease of Pin1 protein	ROS generation	Generation of byproduct
P1D-34	Decrease of Pin1 protein		PROTAC

## Data Availability

No new data were created or analyzed in this study.
